# Coincident choroid plexus carcinoma and adrenocortical tumor in an infant

**DOI:** 10.1007/s10014-012-0112-2

**Published:** 2012-07-01

**Authors:** Kazuhiko Yoshida, Kazufumi Sato, Ryuhei Kitai, Norichika Hashimoto, Toshihiko Kubota, Ken-Ichiro Kikuta

**Affiliations:** Department of Neurosurgery, Faculty of Medical Sciences, University of Fukui, 23-3, Shimoaizuki, Eiheiji-cho, Fukui, 910-1193 Japan

**Keywords:** Choroid plexus carcinoma, Adrenocortical tumor, Malignancy

## Abstract

We report a case of a 20-month-old girl with a large choroid plexus carcinoma arising in the left lateral ventricle and an adrenocortical tumor. Following brain tumor resection, the patient was treated with radiation and chemotherapy. The adrenocortical tumor was found with the manifestation of precocious puberty. *TP53* gene mutation (exons 4–10) was not detected in either specimen. The patient had leptomeningeal dissemination and died 26 months later.

## Introduction

Choroid plexus tumors are relatively uncommon intraventricular neoplasms of neuro-ectodermal origin, accounting for less than 1 % of all intracranial tumors. Most cases occur in children under 2 years of age [1, 2]. Choroid plexus carcinoma (CPC) is even rarer, representing no more than 25 % of all plexus tumors [3–7]. Some choroid plexus malignant tumors are associated with adrenocortical tumors [8–11]. We herein report the case of an infant with no known family history of malignancies who presented with two primary tumors, CPC and a benign adrenal cortical adenoma.

## Case report

**Fig. 1:**
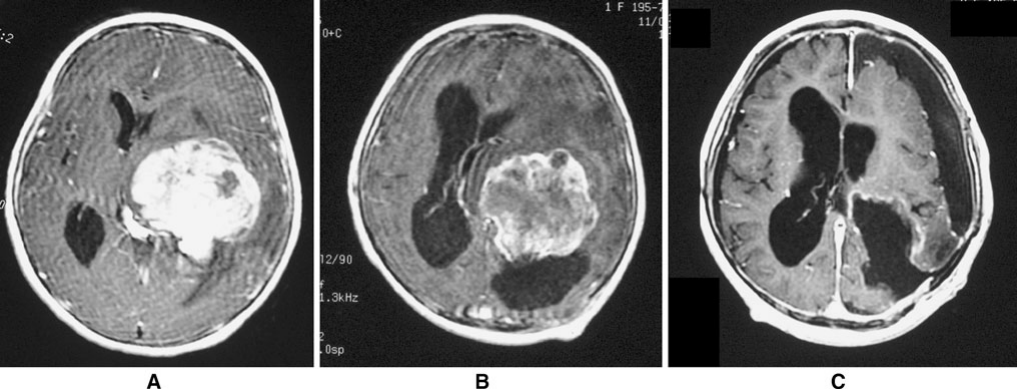
**a** Axial T_1_-weighted magnetic resonance images with gadolinium showing a large tumor in the left lateral ventricle. **b** Gadolinium-enhanced T1-weighted magnetic resonance image following irradiation to the tumor, showing noticeable diminution in enhancement. **c** Postoperative gadolinium-enhanced T_1_-weighted magnetic resonance image showing total tumor excision in the left lateral ventricle

A 20-month-old girl was admitted because of a 3-week history of gradual progression of occasional vomiting and right hemiparesis. Magnetic resonance imaging (MRI) revealed a 7-cm-diameter mass with contrast enhancement in the atrium of the left lateral ventricle (Fig. 1a) and a 1-cm-diameter mass in the interpeduncular fossa. Cerebral angiography revealed a small tumor blush, which was mainly fed by the left anterior and posterior choroidal arteries. The infant’s prenatal and natal histories were of no significance. There was no family history of malignancies. Through left posterio-parietal craniotomy, the patient underwent tumor excision two times during 3 weeks. However, the tumor mass was only partially excised owing to profuse bleeding and brain swelling. For the purpose of reducing the vascularity and size of the tumor, a total dose of 32 Gy of irradiation was given to the limited area of the residual tumor over 4 weeks. The tumor mass regressed on MRI, and the central part of the tumor showed decreased contrast enhancement, which was interpreted as necrosis (Fig. 1b). Subsequently, gross total removal of the tumor in the left lateral ventricle was successfully performed (Fig. 1c), and her right hemiparesis gradually improved. A ventriculo-peritoneal shunt was placed to relieve hydrocephalus.

**Fig. 2:**
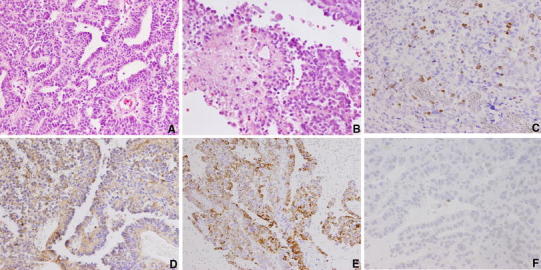
**a** A photomicrograph of the tumor showing papillary and tubular structures lined by single or multiple layered epithelia with hypercellularity. HE stain, original magnification ×200. **b** A photomicrograph of the tumor showing nuclear pleomorphsim and necrotic area. HE stain, original magnification ×400. **c** A photomicrograph of the tumor showing the Ki-67 labeling index is 12 %. Original magnification ×200. **d** A photomicrograph of immunohistochemical staining showing cytoplasmic positivity for transthyretin (TTR). TTR immunoperoxidase, original magnification ×200. **e** A photomicrograph of immunohistochemical staining showing positivity for cytokeratin. Original magnification ×200. **f** A photomicrograph of immunohistochemical staining for p53 showing that few tumor cells were positive. Original magnification ×400

**Fig. 3:**
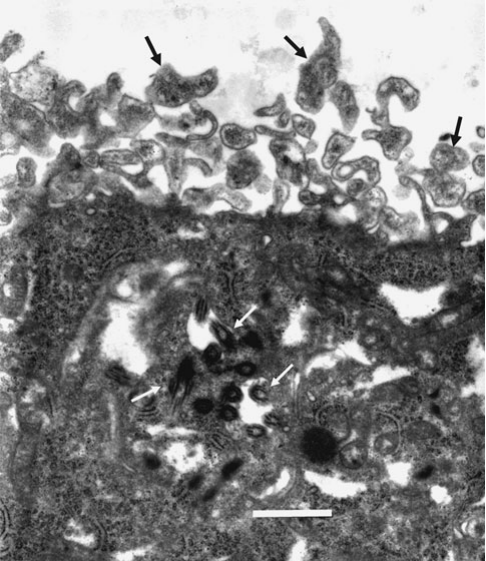
An electron micrograph showing numerous golf-club-shaped microvilli (*black arrows*) on the luminal surface and cilia (*white arrows*) in the cytoplasm. *Bar* 1 μm

Histologically, the surgical specimens in the initial operation were characterized by papillary and tubular structures lined by single or multiple layered epithelia (Fig. 2a). Nuclear pleomorphism and necrosis were found (Fig. 2b). Five or more mitotic figures were seen per 10 HPF (Fig. 2b), and the Ki-67 labeling index was 12 % (DAKO, M7240, Fig. 2c). Immunohistochemical reactivity was positive against transthyretin (DAKO, L1857, Fig. 2d), cytokeratin (45- and 52-kd cytokeratin, YLEM, 5D3, Fig. 2e), S-100 protein (DAKO, Z0311) and vimentin (DAKO, M0725), and was negative for glial fibrillar acid protein (GFAP, DAKO, Z0334), carcinoembryonic antigen (CEA, DAKO, A0115), epithelial membrane antigen (DAKO, M0613) and synaptophysin (DAKO, M0776). Immunohistochemical staining for p53 (DAKO, DO-7) revealed that few tumor cells were positive (Fig. 2f). Electron microscopically, numerous golf-club-shaped microvilli were demonstrated on the luminal surface. Cilia were occasionally seen in the cytoplasm (Fig. 3). A basement membrane was observed on their inner surface. All these histopathological findings were consistent with a diagnosis of CPC.

**Fig. 4:**
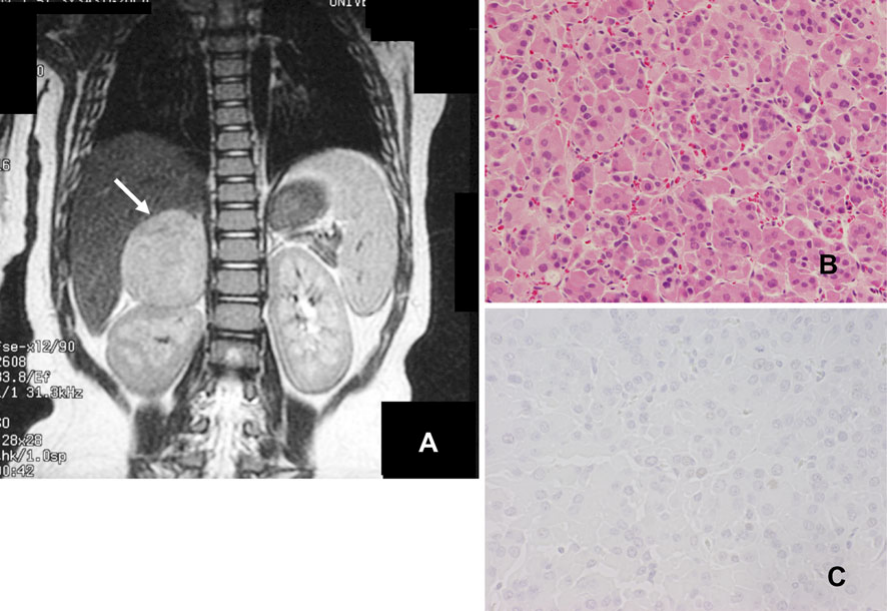
**a** A coronal T_1_-weighted magnetic resonance image showing a large right adrenal tumor (*arrow*). **b** A photomicrograph of the adrenal tumor showing neoplastic growth of atypical eosinophilic cells with a solid or alveolar growth pattern with moderate cellular pleomorphism. No apparent necrosis or invasive growth can be seen. HE stain, original magnification ×200. **c** A photomicrograph of immunohistochemical staining for p53 showing none of the tumor cells was positive. Original magnification ×400

One month after the last operation, the patient developed signs of premature puberty such as pubic and axillary hair growth, hypertrophy of the clitoris and acne. Endocrinological studies revealed exceedingly high values of plasma testosterone at 3.3 ng/ml, the normal level being less than 0.1 ng/ml, and dehydroepiandrosterone-sulfate (DHEA-S) at 21,200 μg/dl, the normal range being 20–119 μg/dl. Abdominal MRI disclosed a large right adrenal mass 5.0 cm in diameter (Fig. 4a). The adrenal cortical tumor was entirely removed, producing immediate resolution of the patient’s symptoms of premature puberty as well as normalization of the plasma levels of testosterone and DHEA-S. Histologically, the tumor consisted of neoplastic growth of atypical eosinophilic cells with a solid or alveolar growth pattern with moderate cellular pleomorphism. Mitotic figures were occasionally seen (1–2 per 10 HPF). No apparent necrosis or invasive growth was detected. Vascular invasion was inconspicuous (Fig. 4b). According to Weiss’s criteria [12], our case was diagnosed as a benign adenoma. None of the adrenocortical tumor cells reacted to p53 antibody (Fig. 4c).

**Fig. 5:**
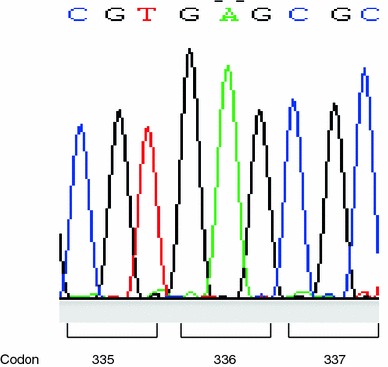
One of the point mutation hotspots of *TP53* gene, codon 337 was judged to have no mutation

Genomic DNA was extracted from the paraffin blocks of the brain tumor and the adrenal tumor (TaKaRa DEXPAT, Takara, Kyoto, Japan), and also from her white blood cells. Amplification of the *TP53* gene within exons 4–10 was carried out with hot-start PCR polymerase (KOD-PLUS, Toyobo, Japan). Sequencing reaction (BigDye Terminator v1.1 Cycle Sequencing Kit, Applied Biosystems) was analyzed using a capillary sequencer (ABI PRISM 3100, Applied Biosystems). No mutation was detected in any of the DNA samples (exons 4–10) extracted from the specimens (Fig. 5).

MRI obtained 5 months after the last operation for the brain tumor disclosed leptomeningeal metastases. After additional radiation therapy and five cycles of chemotherapy including etoposide and carboplatin, tumor regression was observed. Nevertheless, she eventually succumbed to disseminated disease 26 months after the initial surgery.

## Discussion

CPC is the malignant counterpart of choroid plexus tumors and histologically corresponds to WHO grade III [13]. This case fulfilled the following features: frequent mitosis, increased cellular density, nuclear pleomorphism, blurring of the papillary pattern with poorly structured sheets of tumor cells and necrotic area [13]. The differential diagnosis should include papillary ependymoma and metastatic papillary carcinoma, especially from the lung [5, 14]. Immunohistochemistry was positive for transthyretin, cytokeratin, S-100 protein and vimentin in the present case, but there was negative immunostaining for GFAP and CEA. Transthyretin and cytokeratin are reliable markers of choroid plexus neoplasms [15–17]. The expression of cytokeratin and transthyretin by tumor cells and the lack of GFAP expression differentiated this tumor from ependymoma, and the co-expression of cytokeratin, S-100 protein and vimentin with negative CEA was helpful in distinguishing it from metastatic carcinoma [13]. Electron microscopy favored the diagnosis of a choroid plexus tumor owing to the presence of golf-club-shaped microvilli, cilia and a basement membrane [18–20].

CPC in children generally follows an aggressive course [14], and the 5-year survival rate is 26–50 % [2, 3, 5, 6]. The tumor often disseminates via CSF pathways [5, 21], and even metastasizes extraneurally [22]. Previous reports confirm that maximum surgical resection offers the best chance for long-term survival. However, complete surgical excision cannot be carried out in all patients because of the extreme vascularity, larger size and its location [2–6]. To reduce the vascularity and volume of choroid plexus neoplasms, radiation therapy was beneficial in achieving complete resection [23–25].

Childhood adrenocortical tumors (ACT) are very aggressive endocrine neoplasms whose incidence is quite low. According to the International Pediatric Adrenocortical Tumor Registry, they typically present during the first 5 years of life and have female predominance. Hormonal hyperproduction is almost universal, and most patients (84.2 %) present with virilization [26]. Pediatric ACT may occur sporadically or as a component of certain hereditary tumor syndromes, that is, Li–Fraumeni syndrome, multiple endocrine neoplasia type 1, Beckwith-Wiedmann syndrome, Carney complex and congenital adrenal hyperplasia [27].

Sandrini et al. [8] mentioned one pediatric case of combined CPC and adrenocortical carcinoma in 58 cases of childhood adrenocortical tumor. Vital et al. [9] reported a pediatric patient who had adrenocortical carcinoma at the age of 4 years, and the later atypical choroid plexus papilloma was discovered at 6 years, with p53 germline mutation in both tumors. In addition, Wang et al. described a boy aged 18 months who had coincident CPC and adrenocortical carcinoma with elevated p53 protein expression immunohistologically in both tumors [10]. More recently, Russell-Swetek et al. reported a young boy with no family history of cancer who was diagnosed with CPC and adrenocortical carcinoma, and harbored a novel de novo germline *TP53* mutation [11]. Thus, two of these reported cases are considered as Li–Fraumeni syndrome.

Since we failed to show *TP53* germline mutation in our case, this case is likely to be a rare coexistence of CPC and adrenocortical adenoma in an infant. However, both tumors are exceedingly rare; it is possible that the patient had other unknown genetic predispositions toward malignancy.

In conclusion, we should know this type of coincidence of tumors in infants.
